# Comparison of deep learning-based denoising methods in cardiac SPECT

**DOI:** 10.1186/s40658-023-00531-0

**Published:** 2023-02-08

**Authors:** Antti Sohlberg, Tuija Kangasmaa, Chris Constable, Antti Tikkakoski

**Affiliations:** 1 Department of Clinical Physiology and Nuclear Medicine, Päijät-Häme Central Hospital, Lahti, Finland; 2 HERMES Medical Solutions, Stockholm, Sweden; 3 Department of Clinical Physiology and Nuclear Medicine, Vaasa Central Hospital, Vaasa, Finland; 4 Clinical Physiology and Nuclear Medicine, Tampere University Hospital, Tampere, Finland

**Keywords:** Cardiac SPECT, Denoising, Deep learning

## Abstract

**Background:**

Myocardial perfusion SPECT (MPS) images often suffer from artefacts caused by low-count statistics. Poor-quality images can lead to misinterpretations of perfusion defects. Deep learning (DL)-based methods have been proposed to overcome the noise artefacts. The aim of this study was to investigate the differences among several DL denoising models.

**Methods:**

Convolution neural network (CNN), residual neural network (RES), UNET and conditional generative adversarial neural network (cGAN) were generated and trained using ordered subsets expectation maximization (OSEM) reconstructed MPS studies acquired with full, half, three-eighths and quarter acquisition time. All DL methods were compared against each other and also against images without DL-based denoising. Comparisons were made using half and quarter time acquisition data. The methods were evaluated in terms of noise level (coefficient of variation of counts, CoV), structural similarity index measure (SSIM) in the myocardium of normal patients and receiver operating characteristic (ROC) analysis of realistic artificial perfusion defects inserted into normal MPS scans. Total perfusion deficit scores were used as observer rating for the presence of a perfusion defect.

**Results:**

All the DL denoising methods tested provided statistically significantly lower noise level than OSEM without DL-based denoising with the same acquisition time. CoV of the myocardium counts with the different DL noising methods was on average 7% (CNN), 8% (RES), 7% (UNET) and 14% (cGAN) lower than with OSEM. All DL methods also outperformed full time OSEM without DL-based denoising in terms of noise level with both half and quarter acquisition time, but this difference was not statistically significant. cGAN had the lowest CoV of the DL methods at all noise levels. Image quality and polar map uniformity of DL-denoised images were also better than reduced acquisition time OSEM’s. SSIM of the reduced acquisition time OSEM was overall higher than with the DL methods. The defect detection performance of full time OSEM measured as area under the ROC curve (AUC) was on average 0.97. Half time OSEM, CNN, RES and UNET provided equal or nearly equal AUC. However, with quarter time data CNN, RES and UNET had an average AUC of 0.93, which was lower than full time OSEM’s AUC, but equal to quarter acquisition time OSEM. cGAN did not achieve the defect detection performance of the other DL methods. Its average AUC with half time data was 0.94 and 0.91 with quarter time data.

**Conclusions:**

DL-based denoising effectively improved noise level with slightly lower perfusion defect detection performance than full time reconstruction. cGAN achieved the lowest noise level, but at the same time the poorest defect detection performance among the studied DL methods.

## Background

Myocardial perfusion SPECT (MPS) is one of the most common imaging modalities to diagnose coronary artery disease. MPS projection images are inherently noisy because the amount of radiopharmaceutical that is injected into the patient must be limited due to radiation safety concerns. Noise in the projection images progresses to the reconstructed images. This leads to artefacts, which can mimic perfusion defects in the images and can, in the worst cases, lead to misdiagnosis.

The noise level of MPS has been improved by imaging hardware advances, reconstruction algorithm development and by using denoising methods. Cardiac-specific cameras with solid-state detectors have been developed and successfully deployed over the last years. They can be focused to the heart area only and have shown to offer much higher sensitivity than conventional gamma cameras [1]. New organ-specific equipment can be expensive, and thus, attempts to improve the sensitivity of conventional gamma cameras have been made by use of special collimators [2]. In addition to hardware developments, optimized reconstruction algorithms [3] and advanced filtering methods [4] have been utilized to improve the quality of MPS.

Recently, deep learning (DL) methods have entered MPS imaging. They have been demonstrated to improve image noise level, quality and even perfusion defect detection performance [5, 6]. These DL methods are based on training a denoising network with example image pairs consisting of noisy input images and low noise target images. Several different network structures have been presented for cardiac SPECT [5–7], but comparison studies among different networks are lacking. Therefore, the aim of this study was to compare several conventional post-reconstruction convolutional neural networks and to investigate their differences with respect to noise reduction and lesion detection performance. Convolution neural network (CNN) [6], residual network (RES) [7], UNET [8] and conditional generative adversarial network (cGAN) [9] models were implemented. These models were trained using gated clinical MPS studies and different numbers of cardiac gates were summed to generate data with different acquisition times and thus different noise levels.

Denoising easily leads to excessive smoothing of the images [10]. This noise-resolution trade-off must be taken into account when DL denoising methods are evaluated. DL methods are often studied using metrics like signal-to-noise ratio and structural similarity index measure (SSIM), which do take both noise and resolution into account. It is, however, difficult to know how well these metrics relate to clinical tasks. Therefore, in this study, perfusion defect detection performance, which is resolution dependent, was assessed along with the conventional metrics. A receiver operating characteristic (ROC) study based on known perfusion defects added artificially to clinical MPS data was conducted. This study is our first step in a quest to find a clinically meaningful DL method, which would allow significant acquisition time reduction without compromising image quality and defect detection.

## Methods

### Training data for the deep learning models

Training data for the post-reconstruction deep learning models were obtained by randomly sampling 50 stress and rest gated MPS studies (total of 100 acquisitions) from Lahti Central Hospital’s database. The training data included studies reported both as normal and abnormal and were supposed to reflect the entire MPS patient material at our centre. The ethics committee of Joint Authority for Päijät-Häme Social and Health Care has granted approval for this study. The studies were acquired using a 1-day protocol, whereby a vasodilator-induced stress study was performed in the morning and a nitrate-enhanced rest study 3 h later in the afternoon. Weight-based dosing of ^99m^Tc-tetrofosmin was used. The activity of the stress injection was approximately 250 MBq and rest injection 750 MBq. Studies were acquired either with Siemens Symbia T or Siemens Intevo Bold using 90-degree angle between the detectors, 64 projections over 180 degrees rotation, 128 × 128 matrix, 4.8 mm pixel size, 40 s acquisition per projection and 8 cardiac gated frames per R-R interval. The quality of cardiac gating was monitored during acquisition time. Only successfully gated studies were used. After each SPECT study, a low-dose CT was performed using 130 kV tube voltage, 17 mAs tube current and 5.0 mm slice thickness to obtain the attenuation map.

Reduced acquisition time datasets were simulated by summing different numbers of cardiac gates. Full time, half time, three-eighths time and quarter time acquisition data were generated by summing all, half, three and two cardiac gated frames per projection, respectively. The indices (1–8) of the cardiac gates selected for summing for the reduced time acquisitions were randomly sampled for each projection.

All the studies were reconstructed using HERMES Medical Solutions’ (Stockholm, Sweden) HybridRecon ordered subsets expectation maximization (OSEM) algorithm with collimator response, attenuation and Monte Carlo-based scatter modelling [11]. The number of subsets was set to 16, number of iterations 5 and 3D Gaussian post-filter full width at half maximum to 1.25 cm. After reconstruction, images were cropped into 32 × 32 × 32 patches with stride of 8. Image cropping increased the size of training dataset and reduced the memory requirements of the DL model training. Stress and rest patches obtained with half time, three-eighths time and quarter time were all pooled together after cropping and then used to train the DL models with the matching full time patches. Approximately 50,000 patches were used to train each DL model and only one DL model for each DL strategy (CNN, RES, UNET and cGAN) was generated.

### Deep learning models

**Fig. 1:**
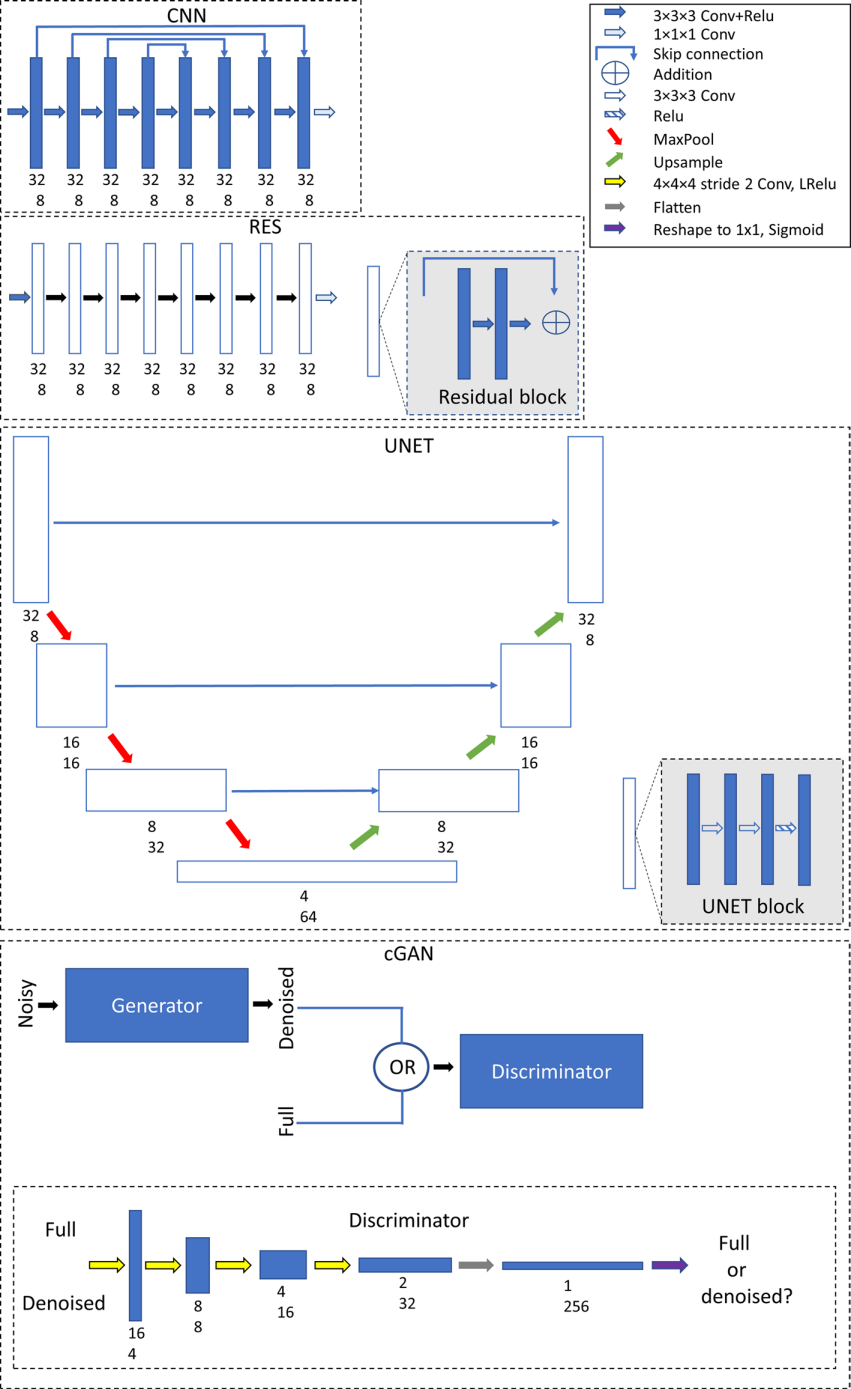
DL models. The number under the blocks presents the patch size (upper number) and number of filters (lower number). Noisy 32 × 32 × 32 patches cropped from reduced acquisition time OSEM images were used as model input and model gave denoised 32 × 32 × 32 patches as output. Output patches were later combined using weighted averaging to produce images at the original reconstruction matrix size

Four different DL-based denoising models were compared. The structure of the models is shown in Fig. 1 in more detail. The CNN model consisted of 8 layers each with 8 filters (3 × 3 × 3 filters and rectified linear unit (Relu) as activation function) and skip connections between the layers. In RES, the convolution blocks of the CNN model were replaced by residual units also shown in Fig. 1. The third model was UNET network. In UNET, the reduced acquisition time patch is first mapped to a latent representation of the input patch in a series of encoding layers. Each resolution level of the encoding path consisted of two convolutional operations followed by Relu (UNET block in Fig. 1). Between resolution levels, the spatial size of the patches is halved using maximum pooling (maxpool) operation. The decoding part, which is used to reconstruct the latent representation into full acquisition time version of the original patch, has similar resolution levels as the encoding path and the patch is up-sampled using transposed convolution between the levels. Skip connections were also used with UNET. CNN, RES and UNET used L2-norm as cost function.

The fourth model was cGAN, which consists of two networks. First network, called generator network, whose structure here is similar to UNET, produces denoised versions of the reduced acquisition time inputs. The second network, called the discriminator, tries to determine if the input is a generator-denoised patch or a true full time patch. The discriminator consisted of 4 convolutional layers, where the image size was reduced with 4 × 4 × 4 stride 2 convolution, followed by a fully connected layer. Leaky Relu (LRelu) was used as an activation function for all other layers except for the last where sigmoid function was used. The cost function for cGAN was a combination of L2-norm for the generator and binary cross-entropy for the discriminator.

The model parameters (number of layers and filters) for all the models were determined by testing different layer and filter number combinations using one-fifth of the training data. Visual image quality and root mean square error with the full time image were used the grade the layer and filter number combinations. The models were generated and trained using Python (version 3.6.8) and Tensorflow (version 2.4). Adam optimizer was applied using the default settings. One hundred epochs with a batch size of 32 were used.

When the DL models were later applied to test data also the test data were cropped into 32 × 32 × 32 patches with stride of 8. The overlapping 32 × 32 × 32 patches were combined after denoising using weighted averaging, where the value of each patch voxel was weighted by the inverse distance of the patch voxel from the patch centre before it was added to the final denoised image. Similar approach was used in [12].

### Testing data for performance assessment

Test data for the DL models were obtained by searching Lahti Central Hospital’s database for stress and rest MPS cases, which the reporting physicians had reported as normal without any visible SPECT perfusion defects. These studies were also not part of the DL training data. Forty-three stress and rest studies were selected. These data were acquired using the same cameras and parameters as the training data. These normal studies were divided into two sets. The first set consisted of 20 studies (20 stress studies and 20 rest studies), which were used to assess the noise level and SSIM and were also used to form studies with known artificial defects. The second set consisted of 23 studies (23 stress studies and 23 rest studies), which were used to build normal databases to assess perfusion detection performance.

For noise level and SSIM assessment, the cardiac gates for the 20 normal studies were summed and full, half and quarter time acquisition data were generated as explained earlier. (Three-eighths time acquisition time data were not used for testing.) All the generated studies were reconstructed using HERMES Medical Solution’s HybridRecon with the same settings that were applied during model training. The four DL-based models were used to denoise the half and quarter time images after reconstruction. Full time, half time and quarter time OSEM without denoising was used as a reference for the DL models.

Perfusion defect detection performance assessment required studies with known defects. Test cases with artificial defects were generated by first reconstructing the 20 stress and rest normal studies using HERMES Medical Solution’s HybridRecon with the same settings that were applied during the DL model training. The studies were then reoriented into short-axis slices and one defect volume of interest (VOI) per normal study with variable size and location was manually drawn on the short-axis images. The defect VOIs were then converted into binary masks and counts in the mask area in the corresponding reoriented normal studies were reduced by 40% and 70%. Total of 40 lesion studies (20 studies, 1 lesion per study and 2 defect percentage levels) were generated for both stress and rest. The lesions were projected and inserted into full time, half time and quarter time acquisition data with an approach similar to the one presented by Narayanan [13]. Finally, the normal full time, half time and quarter time studies and full time, half time and quarter time studies with artificial defects were reconstructed with OSEM using the same settings as previously, post-processed using the 4 DL methods and reoriented into short-axis slices for further analysis.

Perfusion defect detection performance assessment was performed using the total perfusion deficit (TPD) score [14]. TPD is based on comparison to a normal database. The cardiac gates of the acquisition data for the second normal patient set were summed, and full, half and quarter time acquisition data were generated as explained earlier, reconstructed using OSEM, post-processed using the DL models and reoriented into short-axis slices. Six normal databases without DL denoising (stress and rest, full time, half time and quarter time) and four databases per DL denoising method (stress and rest, half time and quarter time) were generated using the Quantitative Perfusion SPECT (QPS) package (Cedars Sinai, Los Angeles, USA).

### Assessment of noise level and SSIM

**Fig. 2:**
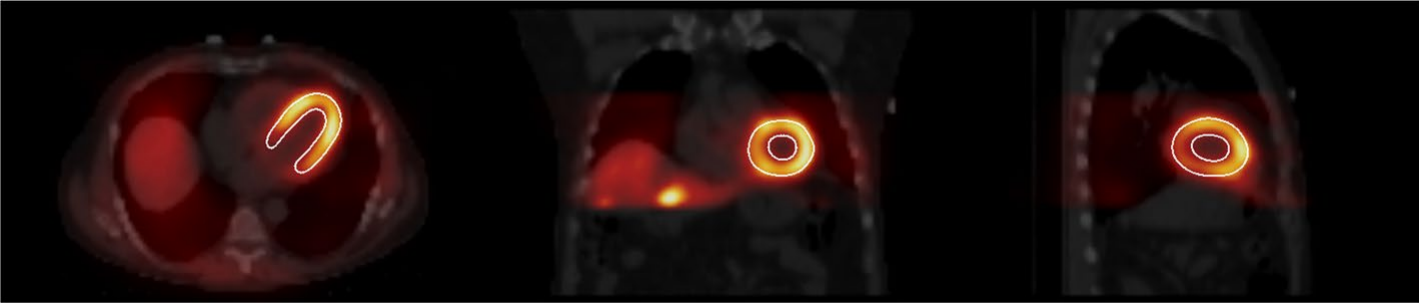
Outlined left myocardium. CoV of counts inside the outline was as a measure of noise. SSIM was also calculated using the outlined myocardium

The left myocardium of the images was outlined with an approach similar to Germano [15]. Coefficient of variation (CoV = 100% × standard deviation/mean) of the segmented left myocardium counts was used as a measure of noise level for the different methods and acquisition times (Fig. 2). SSIM was calculated as

1 $${\text{SSIM}} = \frac{{2\mu_{f} \mu_{r} }}{{\mu_{f}^{2} + \mu_{r}^{2} }}\frac{{2\sigma_{fr} }}{{\sigma_{f}^{2} + \sigma_{r}^{2} }},$$

where subscripts *f* and *r* refer to full time OSEM and reduced acquisition time OSEM or DL methods, *μ* is mean in the myocardium region, and *σ* is variance or covariance. SSIM measures the similarity between two images. The maximum value of SSIM is 1.0, which indicates that two images are identical. Paired t test was used to compare the statistical significance of the CoV and SSIM differences between DL methods and full/reduced acquisition time OSEM without denoising.

### Assessment of perfusion defect detection performance

TPD was calculated for each normal and defect study for each processing method (OSEM, CNN, RES, UNET and cGAN) at each noise level (full time, half time and quarter time) at stress and rest using QPS. TPD scores were used as observer rating for the presence of a perfusion defect. ROC curve analysis was performed based on the ratings and the knowledge of the presence of a defect. Area under the ROC curve (AUC) was used to measure perfusion defect detection performance. ROC curves and AUCs were calculated with MedCalc software (MedCalc Software Ltd, Ostend, Belgium). The statistical significance of the AUC differences between DL methods and full/reduced time OSEM without denoising was tested using the method presented by DeLong [16].

## Results

### Assessment of noise level and SSIM

**Fig. 3:**
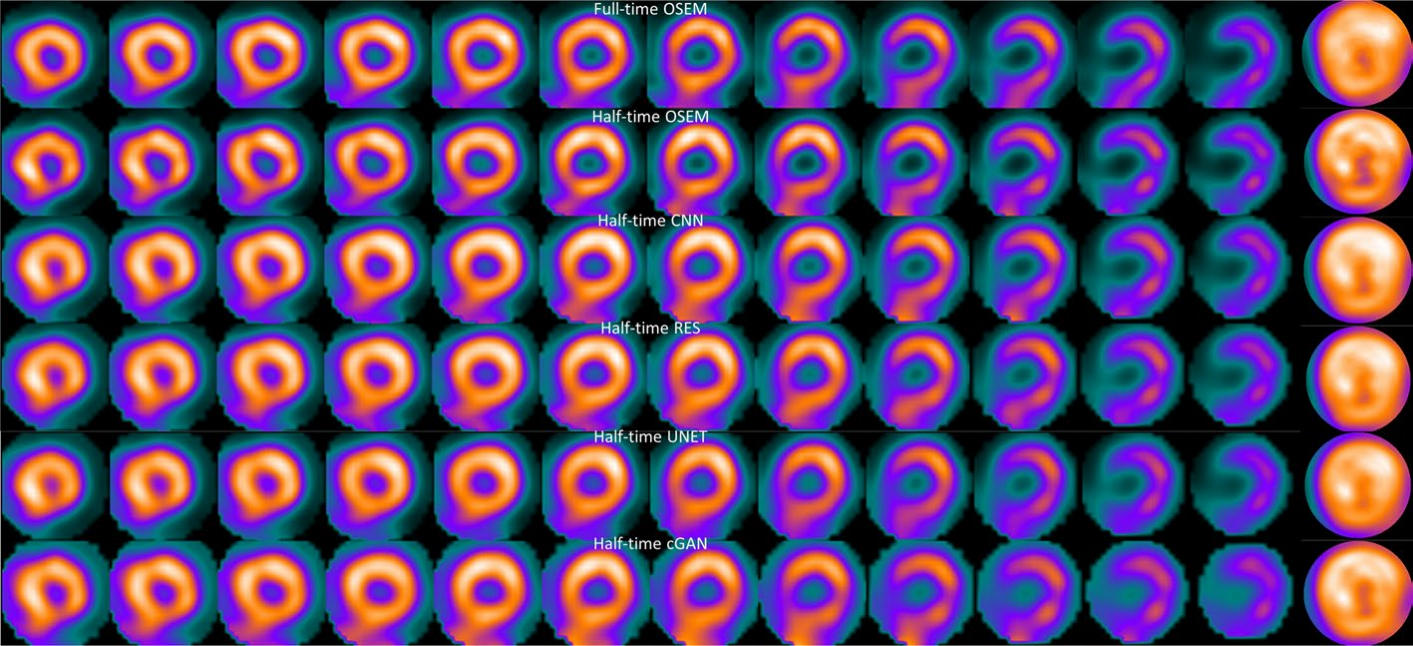
Example half time short-axis slices and polar plot (rightmost column) of a normal stress study

**Fig. 4:**
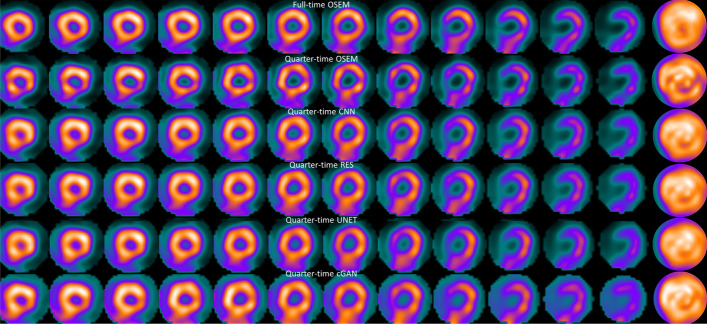
Example quarter time short-axis slices and polar plot (rightmost column) of a normal stress study

Tables 1 and 2 show the CoV for full time OSEM, reduced acquisition time OSEM and DL methods with different acquisition times at stress and rest. The DL methods clearly reduce CoV when compared to reduced acquisition time OSEM without DL-based denoising. This difference is statistically significant (*p* < 0.05) with both acquisition times and stress/rest cases for all DL methods. CNN, RES, UNET and cGAN CoV values with half and quarter acquisition time are even lower than full time OSEM values. The poor performance of OSEM in high noise cases can be seen even more clearly in Figs. 3 and 4, which show example short-axis slices for half and quarter time images for one example patient with normal perfusion. Especially the quarter time OSEM without DL-based denoising image shows artefacts which could be falsely interpreted as perfusion defects. Images processed with the DL have more uniform appearance overall. Tables 3 and 4 present SSIM for reduced acquisition time OSEM and DL methods. OSEM provides overall higher SSIM values than DL methods. This is mainly due to the blurring caused by the DL filtering operations.

**Table 1:** Coefficient of variation (mean ± standard deviation) of myocardium counts for different methods and acquisition times at stress

Acquisition time	Method				
	CoV [%]				
	*p* value versus full time OSEM				
	*p* value versus reduced time OSEM				
	OSEM	CNN	RES	UNET	cGAN
Full	24.8 ± 5.2	–	–	–	–
	–	–	–	–	–
	–	–	–	–	–
Half	25.2 ± 5.3	23.4 ± 5.0	23.2 ± 5.2	23.5 ± 5.3	21.7 ± 5.4
	0.1161	0.0081	0.0010	0.0014	0.0010
	–	0.0002	< 0.0001	< 0.0001	0.0003
Quarter	26.5 ± 4.2	24.1 ± 4.2	23.9 ± 4	24.1 ± 4.5	22.6 ± 4.5
	0.0037	0.2259	0.0744	0.1514	0.0139
	–	0.0002	0.0003	0.0006	0.0008

**Table 2:** Coefficient of variation (mean ± standard deviation) of myocardium counts for different methods and acquisition times at rest

Acquisition time	Method				
	CoV [%]				
	*p* value versus full time OSEM				
	*p* value versus reduced time OSEM				
	OSEM	CNN	RES	UNET	cGAN
Full	21.7 ± 4.0	–	–	–	–
	–	–	–	–	–
	–	–	–	–	–
Half	22.1 ± 4.4	20.7 ± 3.8	20.4 ± 3.8	20.7 ± 4.3	19.0 ± 3.4
	0.1560	0.0184	0.0037	0.0237	0.0011
	–	0.0006	0.0002	< 0.0001	0.0011
Quarter	22.8 ± 3.9	21.1 ± 3.5	20.8 ± 3.5	21.1 ± 4.0	19.5 ± 3.0
	0.0028	0.3314	0.1004	0.2465	0.0068
	–	0.0008	< 0.0001	0.0003	0.0004

**Table 3:** Structural similarity index measure (mean ± standard deviation) of myocardium area for different methods and acquisition times at stress

Acquisition time	Method				
	SSIM				
	*p* value versus reduced time OSEM				
	OSEM	CNN	RES	UNET	cGAN
Half	0.964 ± 0.015	0.944 ± 0.020	0.948 ± 0.020	0.952 ± 0.017	0.923 ± 0.052
		0.0019	0.0037	0.0060	0.0389
Quarter	0.909 ± 0.025	0.911 ± 0.027	0.912 ± 0.025	0.915 ± 0.024	0.890 ± 0.036
		0.7755	0.6148	0.3348	0.1058

**Table 4:** Structural similarity index measure (mean ± standard deviation) of myocardium area for different methods and acquisition times at rest

Acquisition time	Method				
	SSIM				
	*p* value versus reduced time OSEM				
	OSEM	CNN	RES	UNET	cGAN
Half	0.978 ± 0.014	0.950 ± 0.021	0.955 ± 0.018	0.960 ± 0.018	0.936 ± 0.037
		0.0013	0.0005	0.0003	0.0024
Quarter	0.957 ± 0.019	0.941 ± 0.019	0.944 ± 0.014	0.948 ± 0.013	0.921 ± 0.031
		0.0191	0.0046	0.0363	0.0008

### Assessment of perfusion defect detection performance

**Fig. 5:**
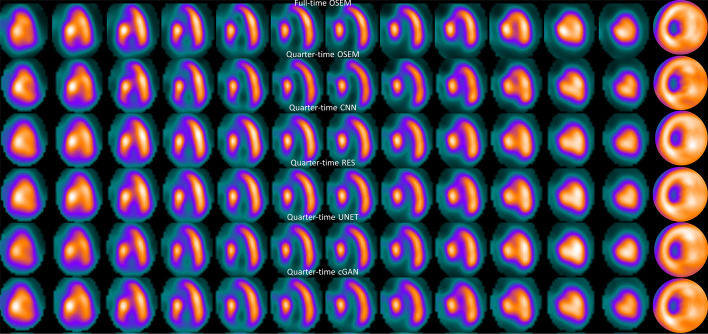
Example quarter time long axis slices and polar plot (rightmost column) of a rest study with 70% deep defect

Perfusion defect detection performance was assessed using the AUC of ROC curve obtained with the different DL methods. All the lesions from different patients, locations and with different depths were pooled and single AUC value for each processing method at each noise level at stress and rest is presented. The AUC values are shown in Tables 5 and 6. DL methods offer comparable, or in some cases even higher, AUC values than reduced acquisition time OSEM without denoising but these differences were not significant, indicating similar performance. Full time OSEM had higher AUC than DL methods. This difference was statistically significant only for CNN, UNET and cGAN in case of rest quarter time study. Example images with artificial perfusion deficit are shown in Fig. 5. The perfusion defect is clearly seen in all images, but the DL images have more uniform appearance than reduced acquisition time OSEM without denoising. The TPD values for this study were 11%, 13%, 10%, 12%, 10% and 12% for full time OSEM, quarter time OSEM, quarter time CNN, quarter time RES, quarter time UNET and quarter time cGAN.

**Table 5:** Area under the ROC curve (AUC ± standard error) obtained with different methods and acquisition times at stress

Acquisition time	Method				
	AUC				
	*p* value versus full time OSEM				
	*p* value versus reduced time OSEM				
	OSEM	CNN	RES	UNET	cGAN
Full	0.97 ± 0.02	–	–	–	–
	–	–	–	–	–
	–	–	–	–	–
Half	0.97 ± 0.02	0.97 ± 0.02	0.96 ± 0.02	0.96 ± 0.02	0.95 ± 0.03
	0.8760	0.9678	0.4779	0.5318	0.3108
	–	0.9300	0.5658	0.6586	0.4207
Quarter	0.93 ± 0.03	0.94 ± 0.03	0.95 ± 0.02	0.93 ± 0.03	0.91 ± 0.04
	0.0673	0.1574	0.3483	0.1539	0.0624
	–	0.4438	0.2722	0.6999	0.5816

**Table 6:** Area under the ROC curve (AUC ± standard error) obtained with different methods and acquisition times at rest

Acquisition time	Method				
	AUC				
	*p* value versus full time OSEM				
	*p* value versus reduced time OSEM				
	OSEM	CNN	RES	UNET	cGAN
Full	0.96 ± 0.02	–	–	–	–
	–	–	–	–	–
	–	–	–	–	–
Half	0.97 ± 0.02	0.94 ± 0.03	0.95 ± 0.02	0.95 ± 0.03	0.93 ± 0.03
	0.7092	0.2773	0.5688	0.3711	0.2358
	–	0.1354	0.2857	0.1083	0.1205
Quarter	0.94 ± 0.04	0.92 ± 0.04	0.93 ± 0.03	0.91 ± 0.04	0.92 ± 0.04
	0.0674	0.0275	0.0554	0.0236	0.0267
	–	0.2889	0.8767	0.2331	0.3744

**Fig. 6:**
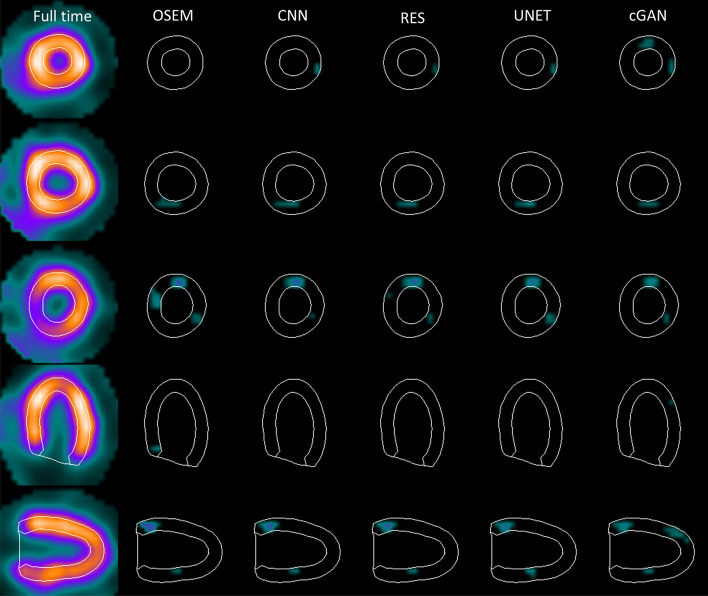
Example difference images obtained with half acquisition time in stress. Full time OSEM image (left column) is shown as reference. The three top rows present short-axis slices at apex, mid-myocardium and base. The bottom two rows show mid-horizontal long axis and mid-vertical long axis images

**Fig. 7:**
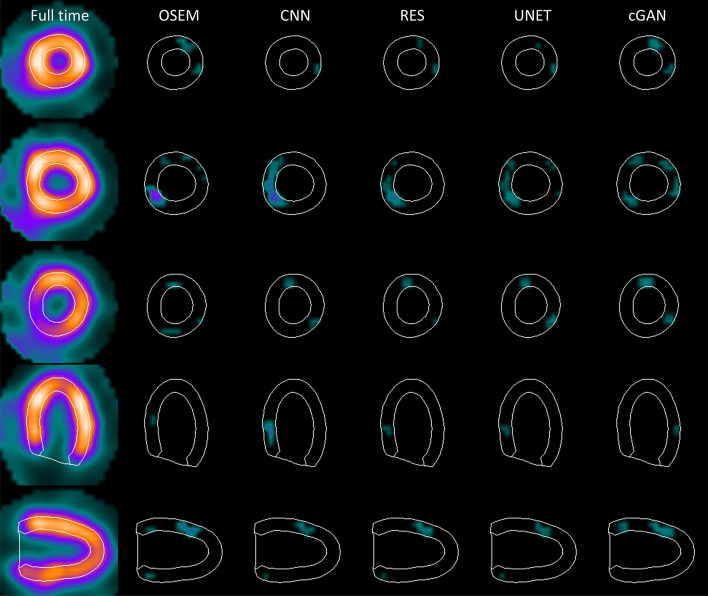
Example difference images obtained with quarter acquisition time in stress. Full time OSEM image (left column) is shown as reference. The three top rows present short-axis slices at apex, mid-myocardium and base. The bottom two rows show mid-horizontal long axis and mid-vertical long axis images

Figures 6 and 7 present example subtraction images where stress half time and quarter time OSEM, CNN, RES, UNET and cGAN images were subtracted from full time stress image. The Compare tool of QPS-package using the Worsening-option was used to generate the images. The differences between full and reduced acquisition time OSEM look deeper overall than the differences between full time OSEM and DL denoising. When acquisition time is reduced to quarter of the original time, the extent of the differences increases for both reduced acquisition time OSEM and DL methods when compared to differences seen in half time images.

## Discussion

Four DL-based denoising methods were compared to full and reduced acquisition time OSEM without denoising. Tables 1 and 2 and Figs. 3 and 4 show that DL methods can achieve substantial noise reduction and improved image quality of myocardial perfusion SPECT studies obtained with reduced acquisition time. This noise reduction happens partly at the expense of perfusion defect detection performance. The area under the ROC curve obtained using the DL methods is comparable to that of reduced acquisition time OSEM without denoising, but poorer than full time OSEM’s. The defects are visually quite clear (Fig. 5).

Differences amongst the more conventional models (CNN, RES and UNET) were small. All the conventional methods performed in a similar manner in terms of noise reduction, perfusion defect detection and image quality with the reduced acquisition time data (Tables 1, 2 and 5, 6, Figs. 3, 4 and 5). Interestingly, the noise reduction performance of cGAN was somewhat better than the performance of other DL methods. Contrary findings have been presented in earlier studies with PET [12, 17], where cGAN did not outperform UNET. The drawback of cGAN is the computationally heavy training process due to more complicated network structure. cGAN also had overall lower AUC values than CNN, RES and UNET (Tables 5 and 6).

The DL methods presented in this work still suffer from resolution-noise trade-off which manifests as blurring of the myocardium seen as low SSIM values in Tables 3 and 4, lower AUC values than full time OSEM in Tables 5 and 6 and visually in Figs. 3 and 4. The DL networks were trained in this study using 32 × 32 × 32 patches that covered the entire reconstruction area. In addition, training data were obtained by pooling all acquisition times at stress and rest and only one model for each DL strategy was generated. This contradicts the approach selected by Ramon [6] and Liu [18], who extracted patches only in the myocardium area and Ramon also trained acquisition time-specific networks. Acquisition time-specific networks were shown to have better performance than the one-size-fits-all approach used in this study [6]. Extracting myocardium centred and acquisition time-specific patches might also reduce image blurring.

The blurring caused by our patch and acquisition time pooling approach probably also reduced the differences between the studied DL models. The structure of the models varied considerably. CNN and RES operate at the same resolution level as the original noisy and the denoised image during all stages, whereas UNET and cGAN include pooling and resampling steps, which can affect image resolution. The resolution loss is difficult to see visually in images 3, 4, 5, 6 or 7 but UNET and cGAN AUCs in Tables 5 and 6 are overall slightly lower than AUCs for CNN and RES. The skip connections used in UNET and cGAN might improve resolution of the denoised images. UNET and cGAN could benefit from minor model update where maximum pooling is switched to strided convolution and upsampling to bilinear interpolation [19].

Post-processing-based denoising used in this work is not the only DL denoising option available. Sun et al. [20] compared pre-reconstruction and post-reconstruction denoising. They used cGAN-type denoising model and noticed that pre-reconstruction-based denoising outperformed post-reconstruction denoising in terms of image quality of mathematical phantoms. Pre-reconstruction denoising can, however, affect the Poisson-nature of the acquisition data, which might compromise the performance of the maximum likelihood type reconstruction conventionally used in emission tomography. Second interesting alternative to post-reconstruction denoising is to include DL-based denoising into statistical reconstruction [21]. Several different approaches to incorporate DL into statistical reconstruction exist. One of the most straight-forward methods is to use an already trained network as a prior for maximum a posterior type reconstruction [22]. Most of the work combining DL and reconstruction has been conducted in the field of PET, but the methods are directly extendable to SPECT. This is a topic for a future study.

This study was limited by the relatively low number of training and testing cases and the fact that cases were obtained at single institute, using two very similar gamma camera systems and that low noise level studies with artificial defects were simulated not acquired. It would be interesting to investigate how well the DL models used in this work generalize to other institutes and scanners. Efforts were, however, made to increase the generalizability by randomly extracting the training material from our institutes database and by pooling the different acquisition time data as mentioned previously. These approaches increase the variability in patients with different cardiac conditions with MPS data acquired with variable noise levels. In addition, clinically meaningful metrics and software were used to evaluate the DL methods. Unfortunately, this approach makes comparison studies labour-intensive. For example, normal databases have to be manually built for each method at each acquisition time and the manual extraction of the TPD results for all the methods is very time consuming. Low noise levels were simulated by summing different numbers of cardiac gates, because acquiring the same patients with different acquisition times is practically impossible. The cameras used in this study do not provide list-mode data either, which would be ideal for resampling studies at different noise levels. Our approach has its’ limitations, but by randomizing the order which gates were summed we could minimize, e.g. the effects of cardiac motion. Data resampling method similar to ours was recently published and shown to be able to provide reduced acquisition time cardiac SPECT data [23].

## Conclusion

DL-based denoising effectively improved noise level with slightly lower perfusion defect detection performance than full time reconstruction. cGAN achieved the lowest noise level performance among the studied DL methods. Further studies are needed to compare different models in evaluation of real-life perfusion defects and artefacts.

## Data Availability

Please contact the corresponding author for the data used in this manuscript.

## Abbreviations

MPS: Myocardial perfusion SPECT
DL: Deep learning
CNN: Convolutional neural network
RES: Residual neural network
cGAN: Conditional generative adversarial neural network
OSEM: Ordered subsets expectation maximization
ROC: Receiver operating characteristic
TPD: Total perfusion deficit
Relu: Rectified linear unit
LRelu: Leaky rectified linear unit
CoV: Coefficient of variation
QPS: Quantitative perfusion SPECT
AUC: Area under curve
