# Correction: Comparison of deep learning-based denoising methods in cardiac SPECT

**DOI:** 10.1186/s40658-023-00542-x

**Published:** 2023-04-06

**Authors:** Antti Sohlberg, Tuija Kangasmaa, Chris Constable, Antti Tikkakoski

**Affiliations:** 1 Department of Clinical Physiology and Nuclear Medicine, Päijät-Häme Central Hospital, Lahti, Finland; 2 HERMES Medical Solutions, Stockholm, Sweden; 3 Department of Clinical Physiology and Nuclear Medicine, Vaasa Central Hospital, Vaasa, Finland; 4 Clinical Physiology and Nuclear Medicine, Tampere University Hospital, Tampere, Finland

**Correction: EJNMMI Physics (2023) 10:9** 10.1186/s40658-023-00531-0

Following publication of the original article [1], the authors identified an error in Fig. 1. The correct figure is given below.

**Fig. 1:**
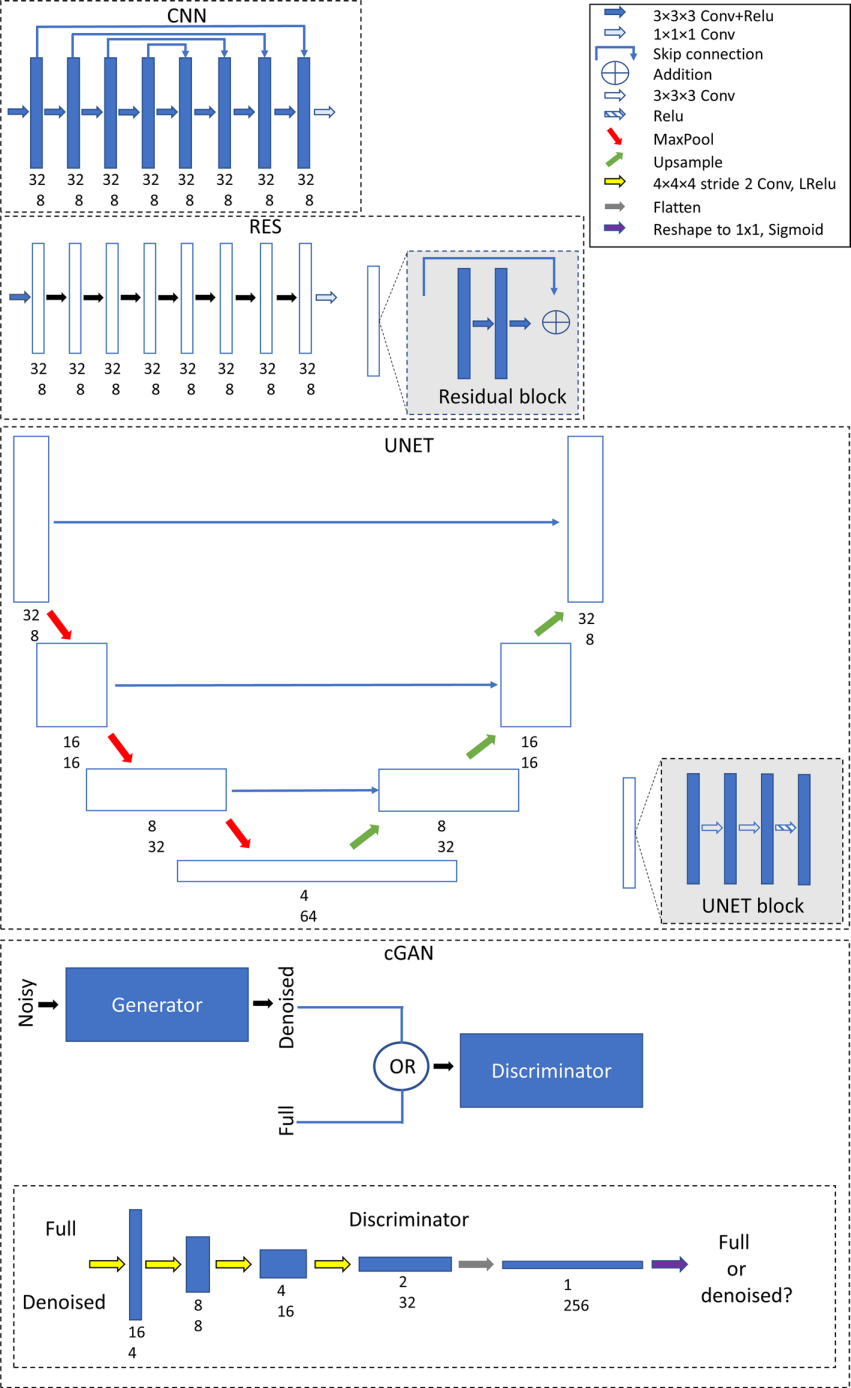
DL models. The number under the blocks presents the patch size (upper number) and number of filters (lower number). Noisy 32 × 32 × 32 patches cropped from reduced acquisition time OSEM images were used as model input and model gave denoised 32 × 32 × 32 patches as output. Output patches were later combined using weighted averaging to produce images at the original reconstruction matrix size

The original article [1] has been updated.
